# Economics of diabetes mellitus: evidence of the disease's social cost for Brazilian data in 2008

**DOI:** 10.1186/1758-5996-7-S1-A204

**Published:** 2015-11-11

**Authors:** Ramon Wiest, Giácomo Balbinotto Neto, Paulo de Andrade Jacinto

**Affiliations:** 1 Universidade Federal do Rio Grande do Sul, Porto Alegre, Brazil

## Background

Diabetes mellitus (DM) is characterized by the high level of blood glucose. Ministry of Health data estimated that Brazil had about 10 million DM cases in 2010, being the fourth main cause of death. World Health Organization estimated the prevalence of DM in Brazil was 10,2% in 2008, about 20 million people.

## Objectives

To measure the DM social cost based in earnings losses of Brazilian workers due to disease in 2008 using data from National Survey of Households of the Brazilian Institute of Geography and Statistics (PNAD/IBGE).

## Materials and methods

A Binary Probit model ws used to measure the participation in work force and a two-stage Heckman model to measure worked h and productivity. Each model is estimated separately for both gender individuals, with and without disease, according three distinct definitions for DM: Restrict, Broad and Comorbidities. To capture the counterfactual effect, the model was calculated for ill and healthy individuals. The difference of both values exhibited the losses, which were aggregate to the whole population and the total cost was estimated.

## Results

According each criterion, respectively, DM reduced the participation in the labor market in 0,97%; 4,60% and 7.06% for men and 0,14%; 4,79% and 6,44% for women, while reduced, respectively 1,51%; 6,40% and 9,15% in productivity and 6,44%; 15,23% and 17,58% in worked h just for women. There was no impact of DM on productivity and in worked h for men. The DM total cost was R$ 8.064 billion, or US$ 3,451 billion converted by current exchange rate. The losses reached 0,73% of total earnings and 0,27% of Brazilian GDP in 2008.

## Conclusions

DM generates significant losses in income of Brazilian workers, especially in relation to their participation in the labor market, since affects both of gender. The results indicate that public policies should be directed to disease diagnosis and prevention, since the development of comorbidities amplifies the effect of losses.

